# PoSE: visualization of patterns of sequence evolution using PAML and MATLAB

**DOI:** 10.1186/s12859-018-2335-7

**Published:** 2018-10-22

**Authors:** Kun Zhao, Elizabeth Henderson, Kelley Bullard, M. Steven Oberste, Cara C. Burns, Jaume Jorba

**Affiliations:** 1 Polio and Picornavirus Laboratory Branch, G-10, Division of Viral Diseases, National Center for Immunization and Respiratory Diseases, Centers for Disease Control and Prevention, 1600 Clifton Rd., N.E, Atlanta, GA 30329 USA; 2 IHRC Inc., Atlanta, GA USA

**Keywords:** Molecular evolution, Bioinformatics, Phylogenetics, MATLAB, PAML

## Abstract

**Background:**

Determining patterns of nucleotide and amino acid substitution is the first step during sequence evolution analysis. However, it is not easy to visualize the different phylogenetic signatures imprinted in aligned nucleotide and amino acid sequences.

**Results:**

Here we present PoSE (Pattern of Sequence Evolution), a reliable resource for unveiling the evolutionary history of sequence alignments and for graphically displaying their contents. Substitutions are displayed by category (transitions and transversions), codon position, and phenotypic effect (synonymous and nonsynonymous). Visualization is accomplished using MATLAB scripts wrapped around PAML (Phylogenetic Analysis by Maximum Likelihood), implemented in an easy-to-use graphical user interface. The application displays inferred substitutions estimated by *baseml* or *codeml*, two programs included in the PAML software package. PoSE organizes patterns of substitution in eleven plots, including estimated non-synonymous/synonymous ratios (dN/dS) along the sequence alignment. In addition, PoSE provides visualization and annotation of patterns of amino acid substitutions along groups of related sequences that can be graphically inspected in a phylogenetic tree window.

**Conclusions:**

PoSE is a useful tool to help determine major patterns during sequence evolution of protein-coding sequences, hypervariable regions, or changes in dN/dS ratios. PoSE is publicly available at https://github.com/CDCgov/PoSE

## Background

Most molecular evolution analysis depends on choosing a model of substitution; for example, to estimate genetic distances or infer a phylogenetic tree. This initial step relies on determining patterns of substitutions, which results in the quantitative analysis of the mutations found in an alignment. Although this may be a relatively quick computational step, understanding how substitutions accumulated and visualizing substitution patterns along the alignment provides a wealth of useful information about the dynamics of nucleotide and amino acid change. There are several approaches to track unique changes along a phylogenetic path. Here, we present work using ancestral reconstruction as implemented in the software package Phylogenetic Analysis using Maximum Likelihood (PAML) [1] for visualizing evolution patterns. The main strengths of PAML lie in the rich repertoire of evolutionary models implemented, which are used to estimate parameters in models of sequence evolution or to test biological hypotheses. Inferred substitutions can be obtained as an optional output in *baseml* and *codeml* programs within PAML, which will generate an additional output file (*rst* file). This file contains the inferred unique substitutions along the phylogenetic tree and is not straightforward to comprehend, particularly for large data sets. In order to visualize the information stored in it, we used MATLAB for capturing, processing, and graphically displaying all changes inferred by PAML. We used this new resource to analyze sequence alignments of rapidly evolving RNA viruses. For example, we examined the pattern of nucleotide and amino acid substitutions to calibrate poliovirus molecular clocks [2] and to define the evolutionary dynamics of circulating vaccine-derived poliovirus (cVDPV) emergences [3].

## Implementation

PoSE is an open source application package with an easy-to-follow graphical user interface (GUI) built in MATLAB. PoSE benefits from MATLAB’s software environment and versatile language syntax for processing large data sets and rendering high-quality graphics. The script also benefits from MATLAB’s extensive development for scientific computing, including applications in bioinformatics and genetic data streamlining (http://www.mathworks.com/company/user_stories/centers-for-disease-control-and-prevention-automates-poliovirus-sequencing-and-tracking.html?by=product). Its GUI was coded using procedural programming, which facilitates addition of future features to the script.

PoSE has over 8,000 lines of MATLAB source code stored in 5 folders and is optimized for MATLAB version 2015b and later versions. PoSE processes the outfile *rst* file generated by *baseml* or *codeml* and produces eleven graphical results and, in addition, interactively displays inferred nucleotide and amino acid substitutions along the phylogenetic tree. The compiled version of PoSE includes all necessary runtime libraries for execution independently from the MATLAB environment. Users do not need MATLAB in order to run PoSE. The compiled version runs in Windows and Mac (10.10–10.13) environments.

The input file for PoSE is the *rst* file generated after running *baseml* or *codeml* in PAML. This file captures the unique nucleotide and amino acid changes along a phylogenetic tree. PoSE requires *rst* files generated from protein-coding sequence alignments free of gaps and ambiguous bases. The user can refer to the PAML manual for addressing questions related to running *baseml* or *codeml* and for treatment of gaps and ambiguities before running PAML.

Each of the eleven plots can be printed or exported as an image in PDF format. In addition, all substitutions displayed in PoSE can be exported in an Excel spreadsheet that includes a Markov matrix of conditional probabilities of observing each type of nucleotide substitution [4]. After reading the *rst* file from *codeml*, PoSE annotates a phylogenetic tree by mapping all nucleotide and corresponding amino acid substitutions occurring in both external and internal branches of the tree. Displayed trees can be exported as an annotated Newick-format file for further inspection using specialized phylogenetic programs such as FigTree (http://tree.bio.ed.ac.uk/software/figtree/).

## Results

**Fig. 1:**
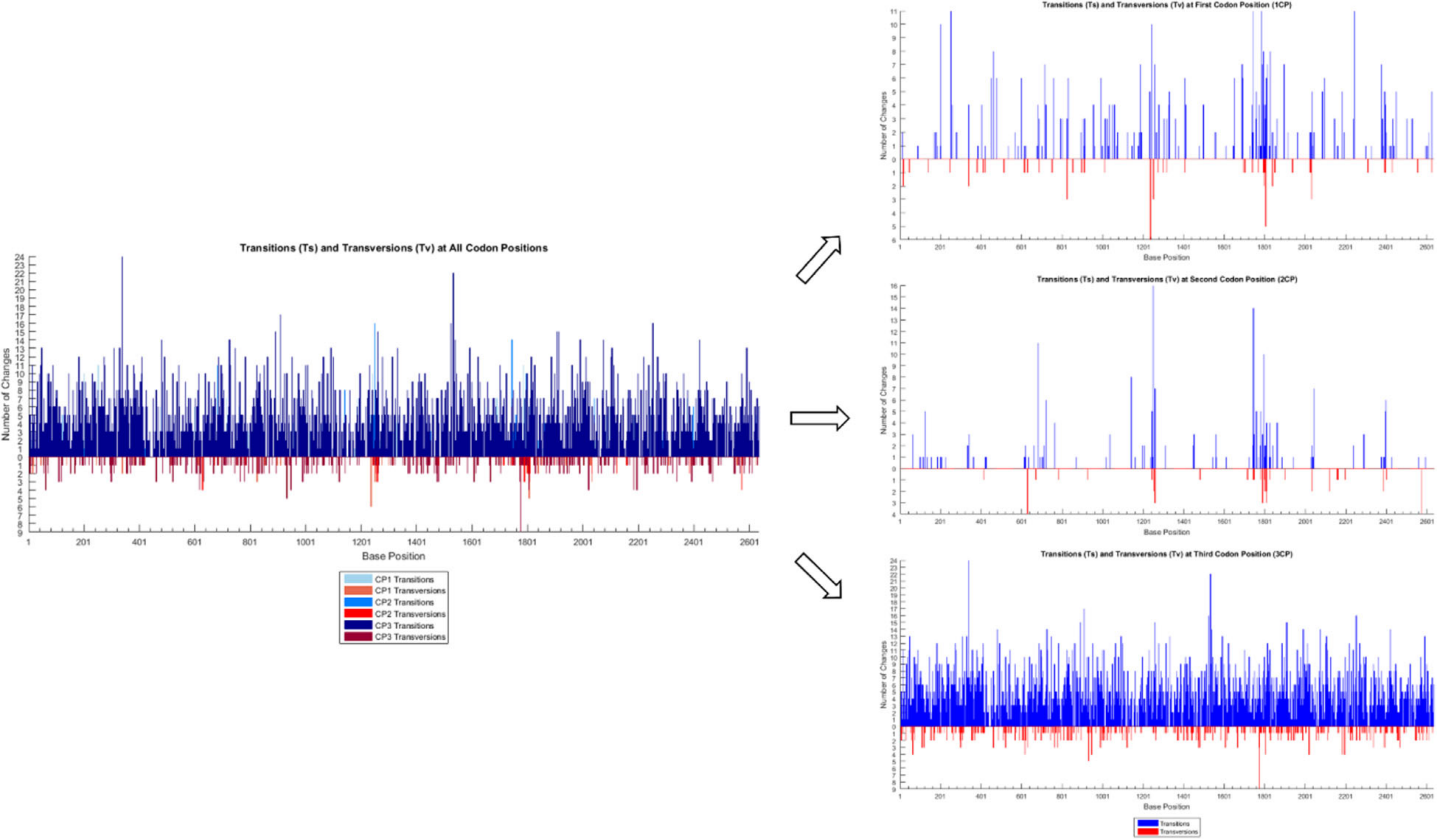
Transition and transversion substitutions (y-axis) at each base position (x-axis) stratified to three codon positions. Data from a survey of wild poliovirus sequences [2]

**Fig. 2:**
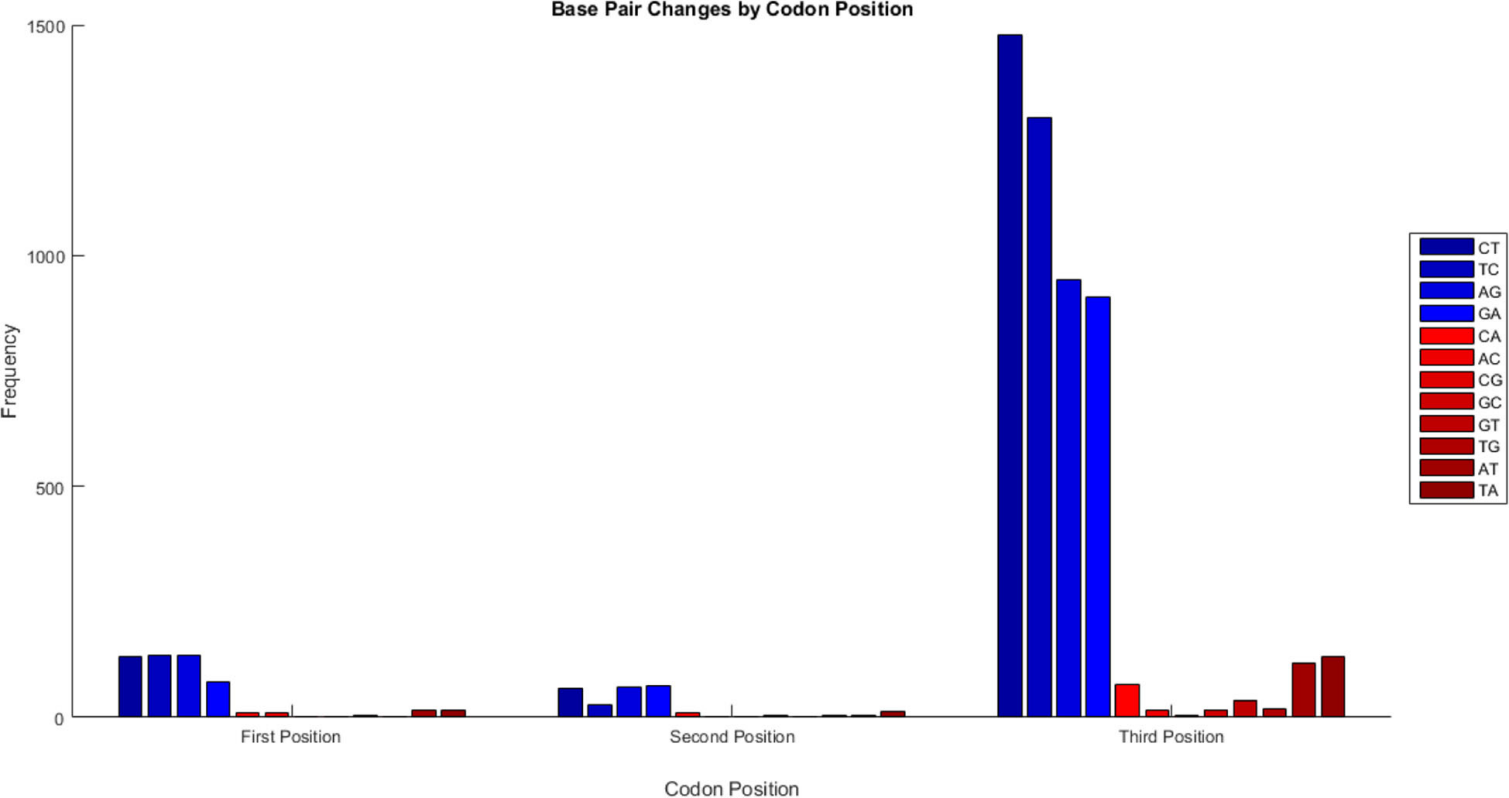
Distribution of all types of transitions and transversion substitutions at each codon position. Data from a survey of wild poliovirus sequences [2]

Visualizations include display of nucleotide and amino acid substitutions occurring along a user-defined sequence interval (summary plots via *baseml*) and along the phylogenetic tree (via *codeml*). Transition (Ts: A↔G, C↔T) and transversion (Tv: A↔C, A↔T, G↔C, G↔T) substitutions are analyzed by codon position (Fig. 1) and then frequency plots summarize the overall accumulation of Ts and Tv and the accumulation of each substitution within transitions and transversions (Fig. 2).

**Fig. 3:**
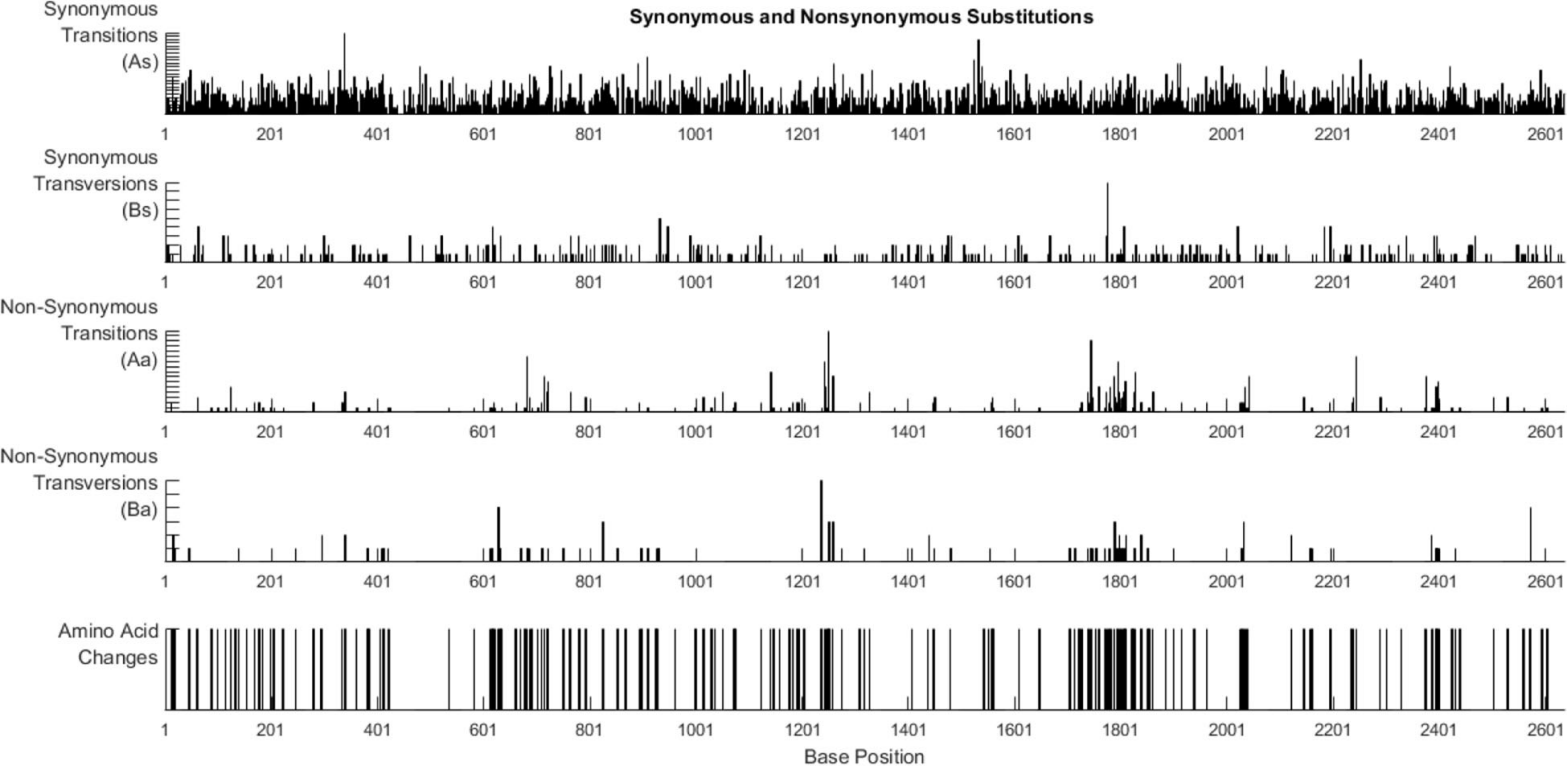
Count of synonymous and nonsynonymous substitutions at each base position. Transitions and transversions were plotted separately. Data from a survey of wild poliovirus sequences [2]

**Fig. 4:**
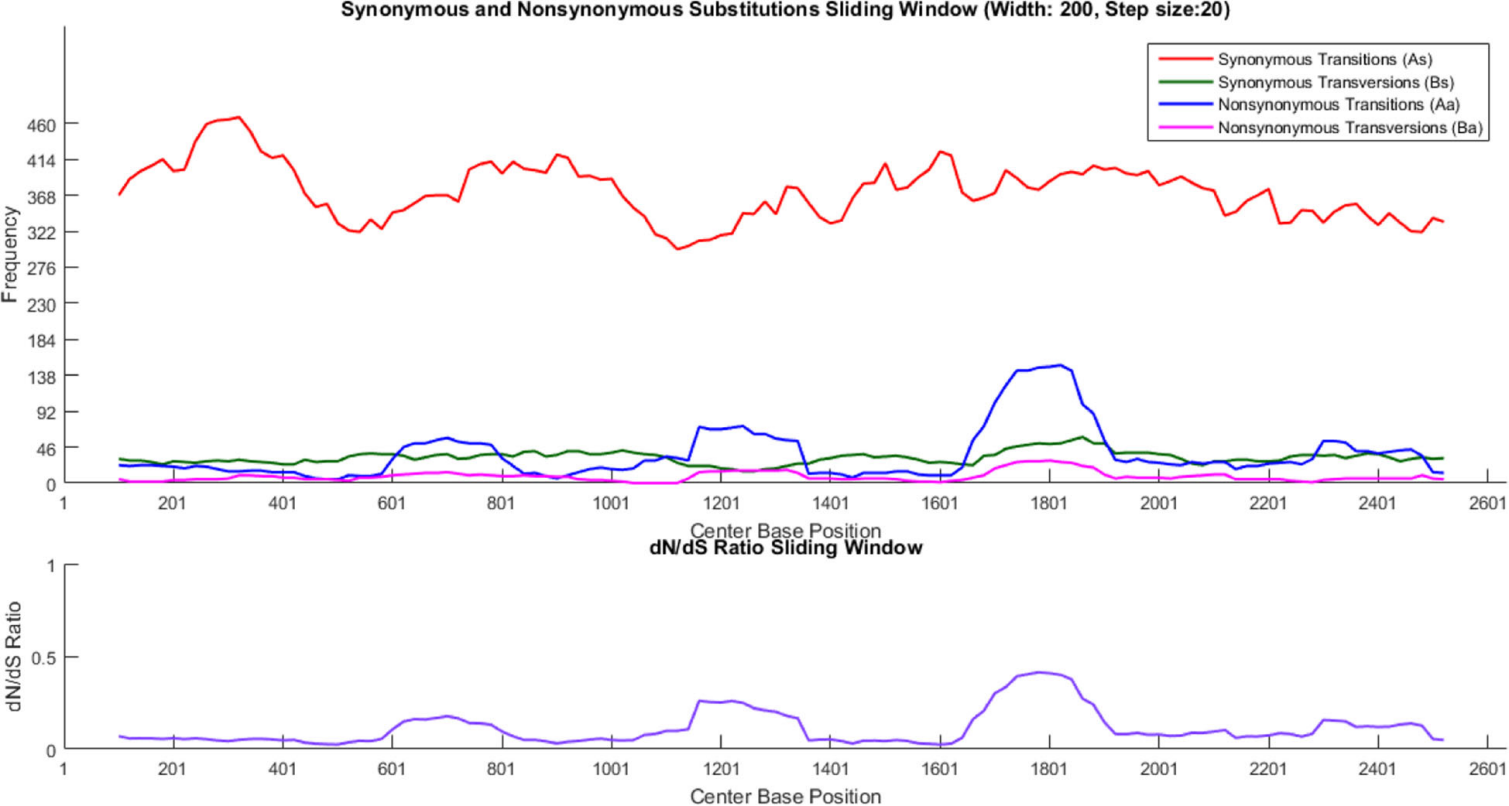
Synonymous substitutions, nonsynonymous substitutions and dN/dS ratio over a sliding window. Upper plot: Dynamics of accumulation of synonymous and non-synonymous substitutions along the sequence interval according to user-defined sliding windows. Lower plot: Estimated dN/dS ratios for each user-defined sliding window. Data from a survey of wild poliovirus sequences [2]

**Fig. 5:**
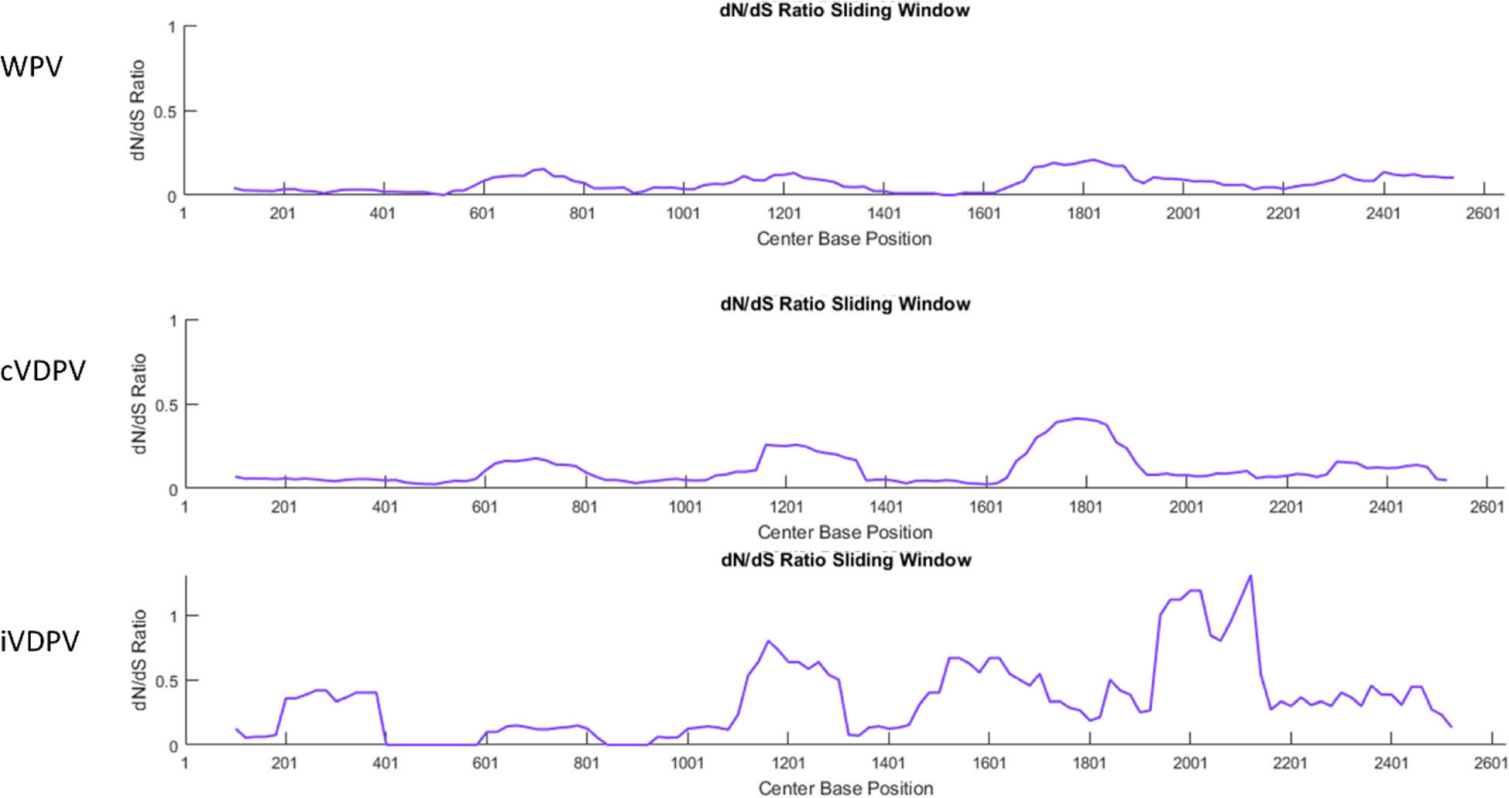
dN/dS ratios estimated from different poliovirus data sets. Upper plot: Wild poliovirus (31 sequences) [2]. Middle: circulating vaccine-derived poliovirus (> 300 sequences) [3]. Lower plot: Immunodeficiency-related vaccine-derived polioviruses (8 sequences) [10]

Phylogenetic signatures along the sequence interval are visualized by inspecting each type of substitution according to the phenotypic effect: 1) substitutions not leading to an amino acid change, synonymous transitions (As) and synonymous transversions (Bs), and 2) substitutions leading to an amino acid change, nonsynonymous transitions (Aa) and nonsynonymous transversions (Ba). Occurrence of these four signals can be graphically visualized at each site along the sequence (Fig. 3). PAML estimates quantities for As, Bs, Aa, and Ba according to the nucleotide evolution model set in the control file. PoSE extracts estimated substitutions and calculates the average of these estimations on user-determined sliding sequence window intervals at shifting step sizes. Total synonymous substitutions (dS = As+Bs) and total nonsynonymous substitutions (dN = Aa+Ba) are calculated for dN/dS ratio. The distribution pattern of As, Bs, Aa, Ba, and dN/dS ratio is summarized in subsequent plots (Fig. 4). Sequence windows and step sizes can be dynamically changed in order to refine the plots or explore different parameters. Likewise, the dN/dS ratio is plotted for identifying sequence regions under putative selection (Fig. 5). The last two plots displayed in PoSE show the cumulative number of As, Bs, Aa, Ba, and total number of substitutions (Kt) along the sequence region in user-specified step sizes.

**Fig. 6:**
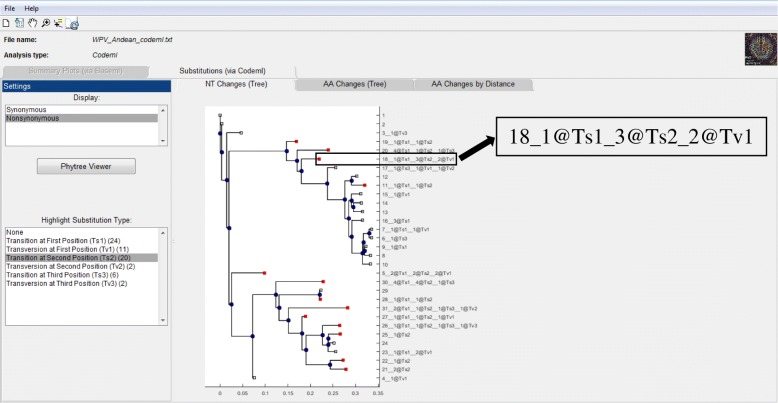
Annotation of a phylogenetic tree with inferred substitutions in external and internal branches. The sequences that have transitions in second position are highlighted in red. For example, the nucleotide substitutions that lead to nonsynonymous mutations are reported as “18_1@Ts1_3@Ts2_2@Tv1”. This means that sequence #18 has one transition (Ts) at the first codon position, three Ts at the second codon position and two transversions at the first codon position

Inferred nucleotide and amino acid substitutions along the path of the phylogenetic tree can be inspected by processing PAML’s codeml results (Fig. 6). In order to visualize the evolutionary pattern calculated from PAML, the tree is annotated with inferred substitutions as synonymous and nonsynonymous substitutions are displayed separately. The program offers the option of highlighting the substitution types inferred at the tips of the tree. The interactive menu allows further tracking of the substitution pattern along the tree; for example, tracking synonymous transitions in particular branches or exploring nonsynonymous substitutions for particular amino acid changes within a branch.

PoSE exports two annotated phylogenetic trees in Newick format; one with inferred amino acid changes and another with corresponding nonsynonymous nucleotide changes. In addition, PoSE generates two reports in Excel format documenting all the data displayed in the plots and in the phylogenetic tree, including a Markov matrix of relative frequencies of specific base changes.

## Discussion

PoSE is a new user-friendly MATLAB script which organizes and graphically displays data from results obtained in *baseml* and *codeml* included in the software package PAML. PAML can be run natively using the command-line or using a GUI interface [5]. PoSE can quickly process large data sets (> 1,000 recorded substitutions). However, prior knowledge of the methods used in these two programs is highly recommended. For example, gaps or ambiguities in protein-coding sequence alignments may produce out-of-frame results. Future versions of PoSE will incorporate a single pipeline from PAML to PoSE, including scanning for potential gaps, ambiguities, or misalignments.

There are numerous software packages and bioinformatics resources for estimating genetic distances and inferring phylogenetic trees from sequence data. However, little is known about the nature and dynamics of accumulation of mutations over a sequence region. PoSE provides visualization of the actual changes occurring in phylogenetically related homologous sequences. For example, inspection of transition and transversion changes per site provides information about patterns in the mode of evolution [6, 7]. Also, graphical visualization of current changes provides a higher level of granularity than that observed in sequence alignments; such as detection of hypervariable regions due to increased number of non-synonymous substitutions (Fig. 3).

The molecular clock model of evolution is of particular interest in studies related to rapidly evolving viruses. Inference from sequence data of the tempo and mode of virus transmission can be readily determined using well-known bioinformatics tools such as BEAST [8]. However, mutation saturation due to multiple substitutions per site can underestimate divergence dates [9]. By analyzing all unique nucleotide substitutions, PoSE scans for putative regions of sequence saturation and provides clues about potential substitutions involved in mutation saturation (Fig. 3).

## Conclusions

PoSE is an ongoing bioinformatics project aiming at analyzing patterns of sequence evolution in protein-coding sequence alignments. It was developed from studies of large data sets of poliovirus genomes. Compatible with the rapid nature of RNA virus evolution, PoSE facilitates processing and visualization of very large data sets containing thousands of inferred nucleotide and amino acid substitutions. PoSE complements inference of genetic distances and phylogenetic trees by contributing detailed information about the nature, distribution, and dynamics of mutations in easy-to-grasp graphical representations.

## Availability and requirements

**Project name:** PoSE.

**Project home page:** https://github.com/CDCgov/PoSE

**Operating system(s):** Windows and Mac (10.10–10.13).

**Programming language:** MATLAB.

**Other requirements:** MATLAB Runtime for free execution of PoSE.

**License:** GNU GPL.

**Any restrictions to use by non-academics:** none.

## Abbreviations

Aa: nonsynonymous transitions
As: synonymous transitions
Ba: nonsynonymous transversions
BEAST: Bayesian Evolutionary Analysis by Sampling Trees
Bs: synonymous transversions
cVDPV: circulating vaccine-derived poliovirus
PAML: Phylogenetic Analysis using Maximum Likelihood
PoSE: Visualization of Patterns of Sequence Evolution
